# Editorial: Integrating technology into advanced Parkinson's disease management: from screening and evaluation to treatment and prevention

**DOI:** 10.3389/fneur.2025.1712760

**Published:** 2025-10-27

**Authors:** Onanong Phokaewvarangkul, Jinyoung Youn, Vijayashankar Paramanandam, Marina Picillo

**Affiliations:** ^1^Chulalongkorn Centre of Excellence for Parkinson's Disease and Related Disorders, Department of Medicine, Faculty of Medicine, Chulalongkorn University and King Chulalongkorn Memorial Hospital, Thai Red Cross Society, Bangkok, Thailand; ^2^Department of Neurology, Samsung Medical Center, Sungkyunkwan University School of Medicine, Seoul, Republic of Korea; ^3^Neuroscience Center, Samsung Medical Center, Seoul, Republic of Korea; ^4^Department of Neurology, Apollo Hospitals, Chennai, India; ^5^Department of Medicine, Surgery and Dentistry ‘Scuola Medica Salernitana', University of Salerno, Salerno, Italy

**Keywords:** Parkinson's disease, management, screening, treatment, prevention

Parkinson's disease (PD) is one of the most common neurodegenerative disorders, characterized by various motor and non-motor symptoms that profoundly affect the quality of life of patients (1). With the rise in global PD prevalence due to aging populations and because of the possibility of neuroprotective therapy in the future, there is an urgent need for innovative approaches to improve PD diagnosis and management. However, the diagnosis and treatment in conventional methods are often limited because they mainly rely on physician assessment and recognition of patient symptoms, which are usually profound at the late stage. Therefore, advanced technologies integrated with digital health platforms, including telemedicine, health applications, wearable devices, and big data and artificial intelligence, offer the ability for early detection, precise evaluation, and ongoing management of PD (2, 3). These technologies represent a new paradigm in PD care.

This Research Topic focuses on the role of advanced technologies in managing PD, from screening and evaluation to treatment and prevention. Its objective is to consolidate cutting-edge research that demonstrates how technology can resolve current gaps in PD management and pave the way for future care of PD patients. It provides a comprehensive overview of the state of the art in technological innovations, highlighting their practical uses, challenges, and future directions in PD management. Ultimately, we include five papers that align with this scope, which are summarized below.

The first paper is from Merlo et al.. The main focus of this study is to systematically review the use of static posturography in assessing balance in patients with Parkinsonism, identify gaps in protocol rationale and sample inclusion, and provide recommendations for future studies to enhance clinical transferability. From 132 studies, 115 studies focused on PD patients and 17 focused on patients with atypical Parkinsonism (MSA, PSP). A total of 4,262 patients were assessed using static posturography. This study found that the domains “transferability to clinical practice” and “assessment protocol” received the lowest scores, indicating a significant gap in current research that still lacks a specific protocol and proper posturography parameters. Therefore, future studies should address these gaps by providing clear protocols, detailing inclusion criteria, and reporting technical information to enable replication.

The second paper is from Durmaz Celik et al. The main focus in this study is to evaluate the compliance and accuracy of the digital diary “MyParkinson's” compared to traditional paper diaries for tracking motor symptoms in PD patients. A total of 22 PD patients were included: 11 in the paper diary group and 11 in the digital diary group. The results showed that the digital diary had significantly better compliance and accuracy, demonstrating substantial to almost perfect agreement with clinical examination notes, and that up to 65% of patients preferred the digital diary for follow-ups. Therefore, by minimizing recall bias and reducing data errors, the digital diary represents a valuable tool for the clinical management of PD. This study also suggests that various patient-based questionnaires or scales can be more useful when accessed electronically.

The third paper is from Ota et al. The main focus of this study is to compare the characteristics of the functional gait domains in PD patients with and without lateral trunk flexion (LTF) determined by gait cycles from patients' self-selected walking speed along a walkway with the motion sensor attached outside of the patients' shoes. A total of 58 PD patients were included: 22 in the LTF group and 36 in the non-LTF group. This study demonstrated that patients with PD and LTF exhibited significantly higher gait variability compared to those without LTF. Moreover, abnormal neural networks may underlie the increased gait variability observed in PD patients with LTF. These results suggest that it is essential to improve physical functions by targeting interventions to better control gait variability. Future studies are needed to target therapeutic interventions at this mechanism.

The fourth paper is from Guo et al. The main focus of this study is to employ bibliometric analysis to examine research hotspots and future trends of insomnia in PD patients. This study analyzed a total of 610 publications. It revealed a consistent upward trend in publications, reflecting increasing academic interest in this field, with the United States and China leading in terms of publication volume. In contrast, the United Kingdom leads in terms of the highest average citations per article. Moreover, since 2019, an increasing interest in several keywords has been observed, such as validation, index, and scale, which provides a clearer future direction for upcoming studies. Therefore, emerging future trends highlight increasing attention to validation, index, and scale development, as well as applications of artificial intelligence and personalized medicine.

The fifth paper is from Kamo et al. The main focus in this study is to quantify rehabilitation interventions for patients with Parkinsonism using wearable devices to guide personalized rehabilitation strategies that improve functional outcomes, focusing on results of body metrics such as body surface temperature, calories expended, and activity indices. A total of 49 patients with Parkinsonism were included: 41 with PD, six with progressive supranuclear palsy (PSP), and two with corticobasal syndrome (CBS). The patients were receiving various rehabilitation therapies, including water stimulation bed, therapeutic massage, sit-to-stand training, gait training, cycle ergometer training, aerobic training using treadmill, stretching, balance training, calisthenics, and resistance training (Kamo et al.). The quantification and visualization of various rehabilitation effects are determined by the therapist using radar charts. Interestingly, rehabilitation using stretching and treadmill techniques showed a significant increase in body surface temperature compared to other methods. These results support the potential benefits of using wearable devices to develop personalized rehabilitation programs for patients with Parkinsonism.

Together, these five papers exemplify emerging technologies in managing PD across diverse areas, including digital tools for symptom monitoring, AI-driven diagnostic support, wearable devices, and specific approaches to enhance clinical decision-making and personalized medicine. These studies highlight the growing role of technology in bridging gaps in PD care, enhancing treatment accessibility, and improving patient outcomes through patient-centered management strategies.
